# Rationale and proposed framework for shared decision making in cardio-oncology

**DOI:** 10.1186/s40959-021-00118-7

**Published:** 2021-08-23

**Authors:** Sarah C. Hull, Aaron Soufer, Erica S. Spatz, Lauren A. Baldassarre

**Affiliations:** 1 Section of Cardiovascular Medicine, Yale School of Medicine, 20 York Street, New Haven, CT 06510 USA; 2 Program for Biomedical Ethics, Yale School of Medicine, 20 York Street, New Haven, CT 06510 USA; 3 Yale Center for Outcomes Research and Evaluation, New Haven, CT USA

**Keywords:** Ethics, Autonomy, Shared decision making

## Abstract

Physicians have a duty to present diagnostic and therapeutic choices with rational guidance that respects patient values and realizes patient goals. In cardio-oncology, we commonly encounter patients who understandably feel overwhelmed or feel that they have no favorable options, particularly in the context of advanced malignancy. Accordingly, a longitudinal multidisciplinary commitment to shared decision making (SDM) ensures that physicians and patients actively participate in this process to promote the best possible outcomes from the patient perspective. We propose a practical framework for approaching these difficult decisions in cardio-oncology drawing upon our experience in clinical practice.

## Background

With the rising cultural emphasis on individualism, the pendulum of modern medical ethics has swung from beneficent paternalism to favor autonomy as the paramount principle in healthcare delivery. Autonomy hinges on the concept of informed consent, relying upon the physician presenting the risks, benefits, and alternatives of diagnostic or therapeutic options so patients can decide how to proceed. However, if not done well, a strictly autonomous approach can leave patients with complex decisions that they are unprepared to make. Presenting too many choices with too little guidance under the guise of maximizing autonomy does a disservice to patients. Psychologists have described how seemingly unlimited choice architecture is demotivating and dissatisfying for consumers [1]. Furthermore, patients are not simply consumers of medical services; they also elicit advice based on physician expertise. On the other hand, clinicians must be mindful of their own potential bias when counseling patients. When physicians assume too much responsibility for decision-making they risk ignoring patient priorities, but when they assume too little responsibility they risk enabling patients to make insufficiently informed decisions that may actually run counter to patient goals or values.

Shared decision making (SDM) has gained traction as an effective strategy to reconcile clinical complexity with patient autonomy, by supporting patients to make informed autonomous decisions that align with their preferences, values, and goals. Considered the pinnacle of patient-centered care [2], the concept of SDM was introduced in 1972 by Robert Veatch [3], who outlined different models of medical decision making with the physician ranging from an engineer devoid of moral responsibility to a religious figure who robs patients of their own moral autonomy. Veatch proposed a contractual relationship between patient and physician sharing ethical authority and responsibility, postulating this could maintain the moral integrity of both parties with a solid foundation of trust. While this strategy offers broad benefits applicable to many disciplines, it is particularly germane to the field of cardio-oncology given its inherent complexity and high-stakes disease states.

Many practical tools have been developed to operationalize SDM in clinical practice, particularly in cardiology [4]. These tools have traditionally focused on singular decisions such as defibrillator implantation. However, longitudinal care of complex patients often requires repeated shared decisions, both large and small, that may be less amenable to standardized decision tools [5]. Here we propose a practical framework for approaching difficult decisions in cardio-oncology, drawing upon our clinical experience in this field. For illustrative purposes, we highlight cases that exemplify principles of SDM in different contexts and stages of care in our cardio-oncology practice. We suggest SDM may be particularly useful in the following situations: 1) when interventions offer unclear benefit and patients feel overwhelmed by cancer treatment, 2) when there is no clear “good” option and patients are forced to choose between the “lesser of two evils,” and 3) when cardiac interventions offer clear benefit in themselves but less so in the context of underlying malignancy.

## When patients feel overwhelmed and interventions offer uncertain benefit

Patients often find cancer treatment overwhelming, requiring frequent encounters and imposing discomfort. Some prioritize maximizing potential benefit by adopting any intervention offered, while others prefer to minimize all but absolutely necessary interventions and encounters with the healthcare system.

For example, cardio-oncology specialists often receive referrals for vague symptoms such as intermittent palpitations. While an event monitor may be indicated for definitive evaluation, patients who are otherwise asymptomatic from a cardiac standpoint with normal electrocardiogram and echocardiogram are likely at low risk for significant cardiac pathology. Whether to proceed with rhythm monitoring that many patients find cumbersome or irritating but others find comforting or reassuring is a shared decision that should be discussed on an individual basis. Similarly, patients may be reluctant to augment an already extensive medical regimen. While they should be strongly encouraged to initiate antihypertensive therapy to counteract VEGF inhibitor-associated hypertension to prevent cardiotoxicity [6], the indication may be less strong to initiate statin therapy for borderline elevated lipids in which case it may make more sense to pursue aggressive lifestyle interventions following completion of cancer therapy. The magnitude of benefit versus tradeoffs of intensifying preventive therapies (such as care burden and medication side effects) must be made clear when deciding on best treatment approaches.

In recent years, the high frequency with which screening echocardiography is recommended with potentially cardiotoxic chemotherapy has also been called into question. In addition to the inconvenience and financial burden that can result from frequent cardiac imaging, it may confer more substantive risks in certain contexts. Specifically, interruption of trastuzumab for the treatment of breast cancer has been associated with decreased recurrence-free survival [7]. As concern for cardiotoxicity is a common reason for trastuzumab interruption, there is ongoing debate in the cardio-oncology community about whether guidelines should relax serial imaging recommendations for low-risk patients such as those without traditional cardiovascular risk factors and without prior anthracycline exposure, especially given that in practice clinicians already demonstrate limited adherence to guideline-recommended screening [8, 9]. This has been attributed not only to the perceived lack of cardiovascular benefit in lower risk patients but also to the potential for harm in the form of worse cancer outcomes if trastuzumab is interrupted for subclinical cardiotoxicity alone [10]. It is not being suggested that current expert consensus and guidelines should not be followed, but rather that shared decision making should also occur with these patients and that their preferences and goals of care should be considered in the approach to cardiotoxicity surveillance. Furthermore, this may require interval reassessment in light of dynamic changes in patient scenarios, such as changes in cancer prognosis which may affect patients’ goals of care. Decisions about imaging frequency were also addressed at the onset of the COVID-19 pandemic, when the benefits of screening needed to be weighed against the risks of exposure before rapid testing, personal protective equipment, and vaccines became widely available [11]. Pending further outcomes data to help refine risk assessment and develop more tailored guidelines for screening algorithms, there is a particularly salient role for shared decision-making in this domain when the risk/benefit ratio remains unclear.

## When there are no “good” options

Cardio-oncology patients may find themselves “stuck between a rock and a hard place” when risks of withholding therapy are comparable to risks of initiating therapy. Decisions about anticoagulation are often especially fraught, which our team encountered in the case of a patient referred to us with metastatic non-small cell lung cancer. Cardiac magnetic resonance (CMR) imaging demonstrated left atrial invasion of the primary tumor with overlying thrombus for which he was started on enoxaparin. Although the initial strategy of anticoagulation for left atrial thrombus seemed straightforward, it was complicated by risk of hemopericardium from mass infiltration. Discussing this case in cardio-oncology conference, our clinical team felt we would rather experience a fatal bleeding event than suffer a debilitating stroke. However, we must be mindful of our biases as clinicians and present difficult choices to patients with objectivity and sensitivity so they can choose for themselves, as their personal and cultural values may lead them to a different decision. For example, physicians may place a higher value on protecting cognitive function while patients might prioritize avoidance of suffering. After a comprehensive discussion the patient elected to continue anticoagulation, but we changed enoxaparin to apixaban to maximize convenience and minimize discomfort despite the lack of evidence supporting it for this specific indication. His disease progressed despite initiation of chemotherapy with carboplatin and abraxane, and his course was complicated by aspiration pneumonia, recurrent seizures, stress cardiomyopathy, and metabolic encephalopathy. Subsequent chest computed tomography (CT) showed lung mass progression with air in the left atrium, and head CT demonstrated bilateral pneumocephalus. At that point his family affirmed it would be in accordance with his wishes to focus on comfort measures, after which he died peacefully.

## When patients have advanced malignancy

When patients are actively dying from advanced malignancy, withholding aggressive cardiac intervention seems straightforward. However, as the landscape of cancer therapy is rapidly evolving (particularly with the advent of immunotherapy), even patients with metastatic malignancy may have life expectancy well over a year. Still, they may be at higher risk of complication or less likely to benefit from certain interventions. Patients with brain metastases in whom dual anti-platelet therapy poses higher bleeding risk might prefer medical management of coronary artery disease (CAD) over percutaneous coronary intervention (PCI) to treat recurrent stable angina. Similarly, patients with limited life expectancy due to cancer but preserved quality of life except for refractory angina might prefer PCI over bypass grafting for multivessel disease. These decisions are best shared among cardiologists, oncologists, and patients. One-size-fits-all or unilateral decision making runs the risk of either denying patients cardiovascular care that could benefit them if their cancer mortality risk is overestimated, or pressuring them into undergoing cardiovascular interventions that may only put them at risk of further complications while undergoing cancer treatment.

Our team cared for a previously healthy patient who presented with metastatic melanoma for which he was started on combination immunotherapy with a trial regimen of dual immune checkpoint inhibitors (ICIs). He developed epigastric pain which was initially concerning for a gastrointestinal complication with transaminitis, but this ultimately evolved into atypical chest pain with mildly elevated troponin and creatine kinase. Accordingly, he underwent expedited cardiac magnetic resonance (CMR) imaging which demonstrated focal T2-weighted and late gadolinium enhancement abnormalities which, although subtle, did meet Lake Louise criteria for myocarditis [12], and his left ventricular (LV) systolic function remained normal. There was also subtle focal enhancement of the lateral pericardium, supportive of a diagnosis of myopericarditis. In light of the subtlety of the CMR findings and the need for ischemic evaluation in the setting of a positive troponin and chest pain, he subsequently underwent non-invasive workup with coronary computed tomography (CT) angiography. This demonstrated non-calcified plaque in the left anterior descending artery (LAD) with associated severe stenosis suspected visually and confirmed to be hemodynamically significant with a value of 0.5 by CT fractional flow reserve (FFR). Following SDM discussion, the patient was admitted electively for left heart catheterization (LHC) with percutaneous intervention of the LAD. In light of the complex clinical scenario of the subtle yet positive findings of myocarditis on the CMR as well as the severe LAD stenosis, the patient opted for endomyocardial biopsy (EMB) to obtain additional information, which was performed 24 h prior to LHC to minimize bleeding risk and ultimately did not show evidence of myocarditis. His LHC did result in a drug eluting stent of the LAD lesion with subsequent resolution of his chest pain. Additional SDM discussions followed among the patient, his wife, the cardiology team, and the oncology team. Given his symptomatic improvement and the absence of arrhythmia on telemetry as well as LV function that remained normal, his unremarkable endomyocardial biopsy, and the fact that immunotherapy remained the best available treatment by far for his advanced malignancy, he ultimately preferred to prioritize resuming cancer treatment over treating his suspected subclinical myocarditis. This is another theme we often encounter in our clinical practice: while it is appropriate for clinicians to be judicious in minimizing treatments with a high likelihood of harm, patients often are understandably more focused on treating their active cancer and are often willing to accept some risk of a potential future cardiac adverse event in order to receive the cancer treatment with the highest likelihood of response. As such, steroids were withheld despite the usual recommendation to initiate them to treat ICI myocarditis [13]. However, contemporary guidance is now available from the American College of Cardiology to assist in gradation and management of varying presentations of ICI associated myocarditis, providing an additional resource for SDM with these patients (https://www.acc.org/-/media/Non-Clinical/Images/Tools-and-Practice-Support/Information-Graphics/2021/02/W21000-CV-Team-Infographics_ICI-pdf.pdf). In our patient, immunotherapy was held for 6 weeks and then initially rechallenged with a single agent before resuming dual ICI therapy in an effort to minimize further cardiovascular injury. He was offered a home rhythm monitor for further risk stratification which he declined. While his troponin never normalized, supporting the diagnosis of subclinical myocarditis, and repeat CMR demonstrated a small pericardial effusion with delayed enhancement, the patient remained clinically stable and asymptomatic from a cardiac standpoint.

## Conclusion

Physicians have a duty to present diagnostic and therapeutic choices with rational guidance that respects patient values and realizes patient goals. Sometimes there is a clear role for intervention with less room for shared decision making, such as PCI for STEMI in an otherwise uncomplicated patient or neurohormonal blockade in a hypertensive patient to mitigate the high potential for cardiotoxicity prior to initiating anthracycline chemotherapy [14]. Still, many decisions do not have such clear-cut “right” answers and require more extensive discussion with direct yet open-ended questions to arrive at a truly shared decision (see Tables 1 and 2). In cardio-oncology, we commonly encounter patients who understandably feel overwhelmed or feel that they have no favorable options, particularly in the context of advanced malignancy. Accordingly, a longitudinal multidisciplinary commitment to shared decision making ensures that physicians and patients actively participate in this process to promote the best possible outcomes from the patient perspective. This commitment should include dedicated education of cardio-oncology fellows in this approach, as well as protecting adequate time during office visits to foster robust and thorough discussions between physicians and patients.

**Table 1:** Questions for patients to consider in cardio-oncology SDM discussions

Are you more worried about your cancer or your heart condition?
Which is most important to you: longevity and survival, functional status and quality of life, or comfort and avoidance of pain/suffering?
Do you prefer a “less is more” approach or rather a “make sure all bases covered” approach – or do you fall somewhere in between?
Do you favor lifestyle interventions to minimize medications, or is it easier for you just to take medications because of other challenging circumstances?

**Table 2:** Questions for clinicians to consider in cardio-oncology SDM discussions

Which poses a greater threat to the patient’s survival and quality of life, cancer or heart disease?
Is long term survival a realistic goal or would a shift in goals of care to focus on quality of life be more successful in your clinical opinion?
What is the risk/benefit ratio of proposed interventions from an oncologic and cardiac standpoint, and how might they conflict with each other?
To what extent might lifestyle interventions supplant the need for pharmacotherapy, and how willing and able might the patient be to adopt lifestyle changes?

## Data Availability

N/A (not original research)
